# ZMPY3D: accelerating protein structure volume analysis through vectorized 3D Zernike moments and Python-based GPU integration

**DOI:** 10.1093/bioadv/vbae111

**Published:** 2024-07-25

**Authors:** Jhih-Siang Lai, Stephen K Burley, Jose M Duarte

**Affiliations:** Research Collaboratory for Structural Bioinformatics Protein Data Bank, San Diego Supercomputer Center, University of California, La Jolla, CA 92093, United States; Research Collaboratory for Structural Bioinformatics Protein Data Bank, San Diego Supercomputer Center, University of California, La Jolla, CA 92093, United States; Research Collaboratory for Structural Bioinformatics Protein Data Bank, Institute for Quantitative Biomedicine, Rutgers, The State University of New Jersey, Piscataway, NJ 08854, United States; Department of Chemistry and Chemical Biology, Rutgers, The State University of New Jersey, Piscataway, NJ 08854, United States; Cancer Institute of New Jersey, Rutgers, The State University of New Jersey, New Brunswick, NJ 08901, United States; Research Collaboratory for Structural Bioinformatics Protein Data Bank, San Diego Supercomputer Center, University of California, La Jolla, CA 92093, United States

## Abstract

**Motivation:**

Volumetric 3D object analyses are being applied in research fields such as structural bioinformatics, biophysics, and structural biology, with potential integration of artificial intelligence/machine learning (AI/ML) techniques. One such method, 3D Zernike moments, has proven valuable in analyzing protein structures (e.g., protein fold classification, protein–protein interaction analysis, and molecular dynamics simulations). Their compactness and efficiency make them amenable to large-scale analyses. Established methods for deriving 3D Zernike moments, however, can be inefficient, particularly when higher order terms are required, hindering broader applications. As the volume of experimental and computationally-predicted protein structure information continues to increase, structural biology has become a “big data” science requiring more efficient analysis tools.

**Results:**

This application note presents a Python-based software package, ZMPY3D, to accelerate computation of 3D Zernike moments by vectorizing the mathematical formulae and using graphical processing units (GPUs). The package offers popular GPU-supported libraries such as CuPy and TensorFlow together with NumPy implementations, aiming to improve computational efficiency, adaptability, and flexibility in future algorithm development. The ZMPY3D package can be installed *via* PyPI, and the source code is available from GitHub. Volumetric-based protein 3D structural similarity scores and transform matrix of superposition functionalities have both been implemented, creating a powerful computational tool that will allow the research community to amalgamate 3D Zernike moments with existing AI/ML tools, to advance research and education in protein structure bioinformatics.

**Availability and implementation:**

ZMPY3D, implemented in Python, is available on GitHub (https://github.com/tawssie/ZMPY3D) and PyPI, released under the GPL License.

## 1 Introduction

Two-dimensional (2D) Zernike moments are mathematical tools used to describe 2D shapes. They have been extensively applied in physics and computer vision (Niu and Tian 2022). Their properties include rotational invariance and orthogonality, enabling facile retrieval of geometric information. Such properties make them efficient and reliable tools for pattern recognition and shape analysis (Niu and Tian 2022). 3D Zernike moments were developed more recently, thanks mostly to the work of Canterakis (1999). They possess similar properties to their 2D counterparts (Novotni and Klein 2003).

Much of structural biology experimental data can be represented as volumetric information (e.g., electron density maps from macromolecular crystallography (MX); electric Coulomb potential maps from 3D electron microscopy (3DEM)). In contrast, atomic level structures are typically represented as point clouds. It is possible, however, to convert point clouds into volumes (e.g. by using Gaussian mixture models (Kawabata 2008)). Thus, most structural biology data are suitable for compact 3D object encoding using 3D Zernike moments, with descriptors independent of rotational pose resulting from rotational invariance.

With the ever-increasing number of experimentally-determined (Burley *et al.* 2023) and artificial intelligence/machine learning (AI/ML)-based predicted structures or computed structure models (CSMs) (Baek *et al.* 2021, Jumper *et al.* 2021), 3D Zernike moments are well-suited to applications in pattern matching and protein structure analysis. For example, 3D Zernike moment analyses have been proposed to help fold classification (Guzenko *et al.* 2020, Aderinwale *et al.* 2022), structural superposition (Ljung and André 2021), protein docking (Venkatraman *et al.* 2009), molecular dynamics simulations (Di Rienzo *et al.* 2020, 2022), structure-based virtual screening (Shin and Kihara 2024), and protein–protein interacting interfaces (Daberdaku and Ferrari 2018, 2019).

Existing 3D Zernike moment calculation methods face challenges in terms of computational infrastructure demands for real-time requirements. Various researchers have focused on improving numerical integration and recursive formulae (Al-Rawi 2012, Hosny and Hafez 2012), but to the best of our knowledge there have been no efforts thus far to implement moment calculation in the popular libraries that support GPU-accelerated computing such as CuPy and Tensorflow. Utilizing GPUs for calculating 3D Zernike moments can be highly advantageous. Parallel processing capabilities can significantly accelerate the computational process. However, current implementations lack effective GPU integration with deep learning frameworks, which do not exploit parallel computing. Doing so should provide advantages when dealing with extremely large quantities of data and/or with the need to generate moments for an arbitrary number of 3D objects. Potentially this could also aid in the efficiency of AI/ML learning processes that utilize 3D Zernike moments.

As 3D Zernike moment calculations are related to spherical harmonics (Hosny and Hafez 2012), vectorizing such mathematical formulae for GPU computing is difficult because they must confront challenges of data dependency and nonlinear computations, including iterative integrals (Schaeffer 2013). Moreover, while calculating the moments, intermediate parameters, such as factorial calculations, must be carefully managed to enhance numerical precision, particularly for higher order Canterakis normalization.

This article presents a new software package, ZMPY3D that supports three Python-based implementations, including NumPy (Harris *et al.* 2020), CuPy (Okuta *et al.* 2017), and TensorFlow (Abadi *et al.* 2016). The package enhances computational efficiency and flexibility, allowing research communities to exploit the power of 3D Zernike moments tool for AI/ML applications and/or algorithm design. The Python package source code is accessible on PyPI and GitHub, allowing installation on diverse platforms, including Google Colab, Linux, and Mac with or without GPU support. Additionally, we provide a tutorial and demonstrations as Jupyter notebooks in the GitHub repository.

## 2 Results

### 2.1 CPU versus GPU performance comparison

Computation times using ZMPY3D in both CPU and GPU environments were evaluated. The analysis was conducted on a personal computer (PC) and in Google Colab; and the testing notebook can be accessed in GitHub repository (see availability). The PC was a Linux system with NVIDIA GeForce RTX 3070 Ti, running Ubuntu 22.04.1 for x86_64 architecture Intel^®^ Core^™^ i7-12700K (12 cores). Google Colab provides GPUs and CPUs, and we tested hardware that use GPUs (Tesla T4, L4, and V100) and CPU Intel^®^ Xeon^®^ E5 v4 CPU family @ 2.20 GHz (2 cores, 55 MB cache). TensorFlow version 2.15.0 and CuPy version 12.2.0 were used for GPU-acceleration. A voxel cube with dimensions of 100 × 100 × 100 was applied to perform 10,000 3D Zernike moment calculations, using two maximum orders (20 and 40). Results are presented in Table 1. The speed-up from our vectorized NumPy (CPU) implementation to our vectorized GPU implementation is in the range of 30× to 100×. Speed-up versus other existing non-vectorized CPU-based implementations is likely to be even higher. For instance, we compared the computation time against the BioZernike library (Guzenko *et al.* 2020), a publicly available Java-based software for calculating 3D Zernike moments. Since BioZernike lacks the capability to explicitly initialize a gridded bounding box, we used a structure, specifically PDB code 1HHS, chain A, with dimensions of 82 × 87 × 81. It should be noted that BioZernike does not facilitate normalization at order 40, nor does it support dynamic loading of pre-calculated caches or any kind of parallel computing such as vectorized GPU operations and CPU multithreading.

**Table 1.** Computation time with ZMPY3D in both CPU and GPU environments.

	Tensorflow				CuPy			
Order	T4	RX3070Ti	V100	L4	T4	RX3070Ti	V100	L4
20	1m1s	0m36s	0m31s	0m39s	4m45s	2m30s	1m42s	2m50s
40	24m40s	9m3s	10m54s	11m13s	35m20s	19m19s	14m45s	18m40s

	NumPy		BioZernike	
Order	CPU1	CPU2	CPU1	CPU2
20	33m20s	14m1s	426m40s	89m50s
40	951m40s	338m20s	N/A	N/A

CPU1 stands for Intel^®^ Xeon^®^ E5 v4 CPU family @ 2.20 GHz, 55MB cache; CPU2 stands for Intel^®^ Core^™^ i7-12700K. A 100³ voxel cube was used for 10 000 3D Zernike moment calculations at maximum orders of 20 and 40.

### 2.2 Structural superposition

Traditional protein structure superposition methods rely on atomic coordinates and frequently require chain connectivity. In contrast, volume-based methods offer several advantages: first, they do not depend on the chain connectivity; second, they are directly applicable to quaternary structure; and third, they can be used for either volumetric data (e.g., 3DEM maps), or atomic coordinates (following a trivial conversion process). One drawback of volume approaches is the fact that they do not provide well-understood metrics pertaining to atomic coordinates such as RMSD.

**Figure 1.**
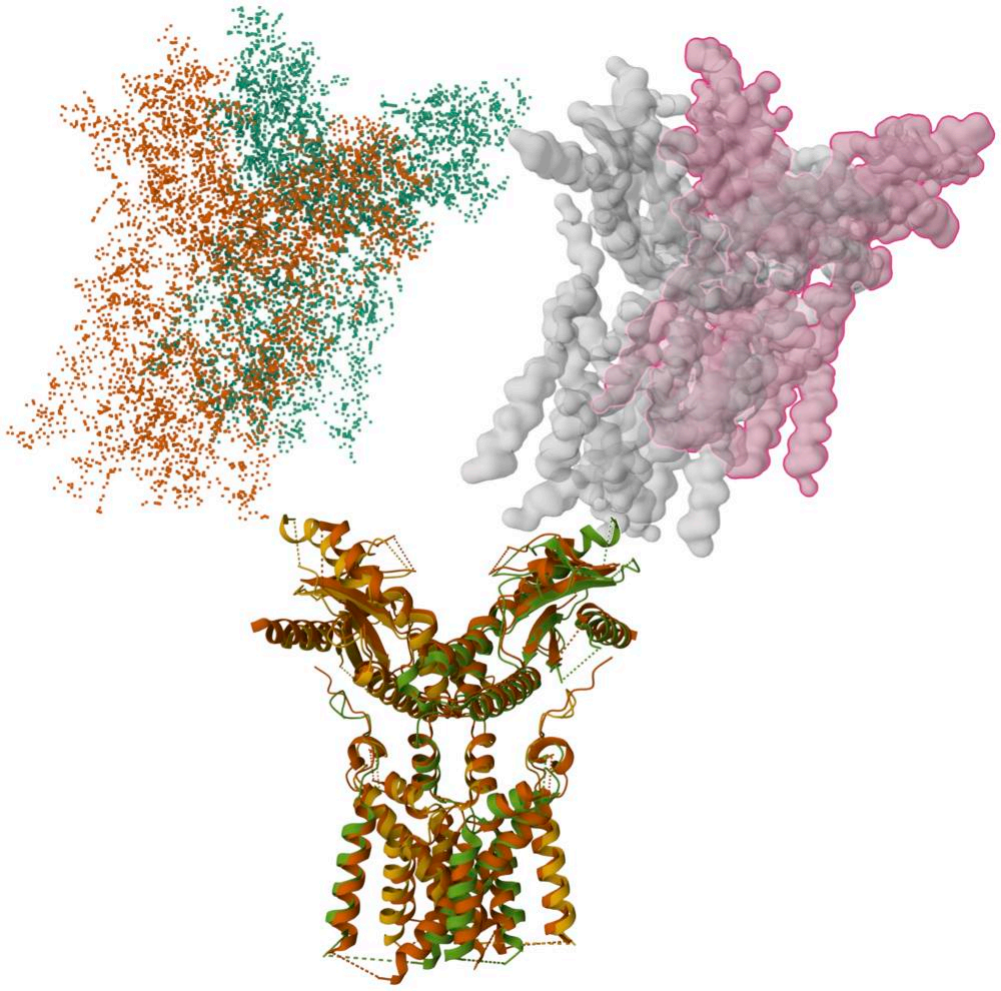
Structural superposition from point clouds to gridded voxels. This figure illustrates the transformation of the atomic coordinates of PDB entries 6NT5 and 6NT6 (top left) into voxels (top right) using the Gaussian mixture model (Kawabata 2008), followed by the computation of two sets of 3D Zernike moments. The transformation matrix for the superposition of the two structures (bottom) is calculated by selecting the pair of vectors with the highest dot product value.

In ZMPY3D, we implemented volume-based structural superposition, following the procedure developed by Guzenko *et al.* (2020). The procedure (shown schematically in Fig. 1) begins with converting atomic coordinates into voxels by placing a Gaussian density feature centered on each C-alpha atom, followed by generation of 3D Zernike moments based on the voxels of each protein. and then normalization of the 3D Zernike moments (Canterakis 1996) to produce alternative moments, a process that yields rotation matrices. The final steps involve computing dot products of all pairs of 3D Zernike moments, selecting moments corresponding to maximum values of the dot products, and using them to derive the transformation matrix. The more efficient implementation introduced here offers the possibility of carrying out protein structure superpositions on much larger datasets.

## 3 Conclusion

In this application note, we present a new software tool that increases the efficiency of computing 3D Zernike moments with vectorization and GPU-computing. The tool should prove useful for many applications, going beyond protein structure bioinformatics. For example, an immediate application is employing ZMPY3D in data loaders for deep learning pipelines to achieve faster data conversion allowing processing of large-scale structure datasets.

More generally, 3D Zernike moments represent a highly versatile tool, providing an effective method for describing 3D volumes and establishing a unified analytical framework for both atomic level structure information and 3D volumetric data. One can transform rich information from geometry, shape, volume, and 3D templates (Riziotis and Thornton 2022) into 3D Zernike moments. Furthermore, AI/ML methods can be applied directly to volumetric 3DEM experimental map data (Maddhuri Venkata Subramaniya *et al.* 2019, Giri *et al.* 2023), wherein utilization of 3D Zernike moments can deliver insights across the biological and biomedical sciences.

The tool presented here enables efficient combination of 3D Zernike moments with modern robust AI/ML models, such as deep convolution neural networks and large language models and thereby deepen our understanding of protein structure and advance research and education in structural bioinformatics.

## Data Availability

The data underlying this article are available in GitHub at https://github.com/tawssie/ZMPY3D.
